# Revisiting the Genetic Ancestry of Brazilians Using Autosomal AIM-Indels

**DOI:** 10.1371/journal.pone.0075145

**Published:** 2013-09-20

**Authors:** Fernanda Saloum de Neves Manta, Rui Pereira, Romulo Vianna, Alfredo Rodolfo Beuttenmüller de Araújo, Daniel Leite Góes Gitaí, Dayse Aparecida da Silva, Eldamária de Vargas Wolfgramm, Isabel da Mota Pontes, José Ivan Aguiar, Milton Ozório Moraes, Elizeu Fagundes de Carvalho, Leonor Gusmão

**Affiliations:** 1 DNA Diagnostic Laboratory (LDD), State University of Rio de Janeiro (UERJ), Rio de Janeiro, Brazil; 2 Institute of Molecular Pathology and Immunology of the University of Porto (IPATIMUP), Porto, Portugal; 3 Laboratory of Human Identification, University of Pernambuco, Pernambuco, Brazil; 4 Institute of Biological Sciences and Health, Federal University of Alagoas, Alagoas, Brazil; 5 Núcleo de Genética Humana e Molecular, Departamento de Ciências Biológicas, Centro de Ciências Humanas e Naturais, Universidade Federal do Espírito Santo, Vitória, Brazil; 6 Laboratório de Diagnóstico Molecular do Centro de Apoio Multidisciplinar, Universidade Federal do Amazonas, Amazonas, Brazil; 7 Department of Internal Medicine, Faculty of Medical Science, Federal University of Mato Grosso do Sul, Campo Grande, Brazil; 8 Laboratório de Hanseníase, Instituto Oswaldo Cruz, FIOCRUZ, Rio de Janeiro, Brazil; University of Utah, United States of America

## Abstract

There are many different studies that contribute to the global picture of the ethnic heterogeneity in Brazilian populations. These studies use different types of genetic markers and are focused on the comparison of populations at different levels. In some of them, each geographical region is treated as a single homogeneous population, whereas other studies create different subdivisions: political (e.g., pooling populations by State), demographic (e.g., urban and rural), or ethnic (e.g., culture, self-declaration, or skin colour). In this study, we performed an enhanced reassessment of the genetic ancestry of ~ 1,300 Brazilians characterised for 46 autosomal Ancestry Informative Markers (AIMs). In addition, 798 individuals from twelve Brazilian populations representing the five geographical macro-regions of Brazil were newly genotyped, including a Native American community and a rural Amazonian community. Following an increasing North to South gradient, European ancestry was the most prevalent in all urban populations (with values up to 74%). The populations in the North consisted of a significant proportion of Native American ancestry that was about two times higher than the African contribution. Conversely, in the Northeast, Center-West and Southeast, African ancestry was the second most prevalent. At an intrapopulation level, all urban populations were highly admixed, and most of the variation in ancestry proportions was observed between individuals within each population rather than among population. Nevertheless, individuals with a high proportion of Native American ancestry are only found in the samples from Terena and Santa Isabel. Our results allowed us to further refine the genetic landscape of Brazilians while establishing the basis for the effective application of an autosomal AIM panel in forensic casework and clinical association studies within the highly admixed Brazilian populations.

## Introduction

Despite the nature of genetic markers (e.g., blood groups, proteins or DNA sequences) or their location in the genome (e.g., mitochondria, autosomes or heterosomes), polymorphisms known as Ancestry Informative Markers (AIMs), present very high intercontinental allelic differentiation across populations (e.g., [1,2,3]).

The investigation of genetic ancestry profiles of human populations is a valuable tool to understand the dynamics of migrations and colonisation events, as well as to determine admixture patterns inside populations.

Ancestry estimates play an important role in correcting for population stratification effects in case-control genetic association studies (e.g., [4,5]), particularly in studies carried out on ethnic admixed individuals in which spurious genotype-phenotype associations may appear due to differences in the allele frequencies of parental groups that contribute differentially in case and control samples. To avoid misinterpretation of the association results, individual ancestry estimates can be considered when calculating statistics (e.g., STRAT software; http://pritch.bsd.uchicago.edu/software/STRAT.html), in addition to pre-selecting criteria to match controls with available cases (for a revision on this subject see 6).

In the field of forensic genetics, having a set of markers that may provide estimates of ancestral membership proportions or help identify the source population of the donor of a certain piece of evidence can help direct the criminal investigation [7,8].

Brazil is well known for the heterogeneous distribution of three main ancestral contributions from Native Americans, Europeans and Africans. These people met and mated among themselves in distinct ways, giving rise to a highly multiethnic admixed population. The European and African colonisation of the Brazilian territory, previously occupied only by Native Americans, started on the coast and gradually reached the interior. The progression of colonisation was highly diverse in different regions, as far as European, African and Native American parentages were concerned [9]. This complex process, in a territory of almost continental dimensions, is consequently reflected in the variance in the genetic composition of the present populations (e.g., [10,11,12]).

The first attempts to evaluate the ethnic diversity of Brazilians were based on blood groups and protein markers and provided a broad overview of the ethnic heterogeneity associated with the different geographic regions of the country (e.g., [13,14]). Since the beginning of its application to population genetics, lineage markers have been used to understand the complex process of admixture and to characterise the mating patterns across the country (e.g., [15,16,17,18,19]). More recently, a series of studies were undertaken using autosomal markers representing different types of DNA variation, namely, Short Tandem Repeats (STRs), Single Nucleotide polymorphisms (SNPs) and Insertion-deletions (Indels) (e.g., [3,11,12,20]).

Apart from the variation in the number and/or type of markers that have been used to evaluate admixture processes in Brazil, consideration of the various sampling strategies that have been used until now is also important. In many studies, it is not possible to have a comprehensive view of the population gene pool because only some population subgroups are investigated, namely, groups classified based on (a) self- reported ancestry, (b) social stratus, (c) skin colour (d) or other phenotypic classifications.

Considering the demography of Brazil in addition to the disparity of markers and sampling criteria that have been used in different publications, the ancestry of the Brazilians is far from fully known. Hence, additional analyses using larger random samples to cover new populations can be helpful in determining novel aspects of the genetic structure within Brazil’s five macro-regions. To accomplish this objective, we performed thorough analyses involving existing data [3,21], the typing of ancestry-informative autosomal Indels (AIM-Indels), and the comparison of our results with those from other publications.

## Materials and Methods

### Ethics Statement

All samples involved in the study were anonymised DNA extracts previously obtained from healthy unrelated individuals. The samples were collected under written informed consent to participate in this study. This study was approved by the Ethic Committees at the State University of Rio de Janeiro (CAAE:0067.0.228.000-09). The current study complies with the ethical principles of the 2000 Helsinki Declaration of the 206 World Medical Association (http://www.wma.net/en/30publications/10policies/b3/). The access to the Terena community living area was approved by FUNAI second authorisation n.º 016/CGEP/99.

### Sample collection and DNA extraction

**Figure 1:**
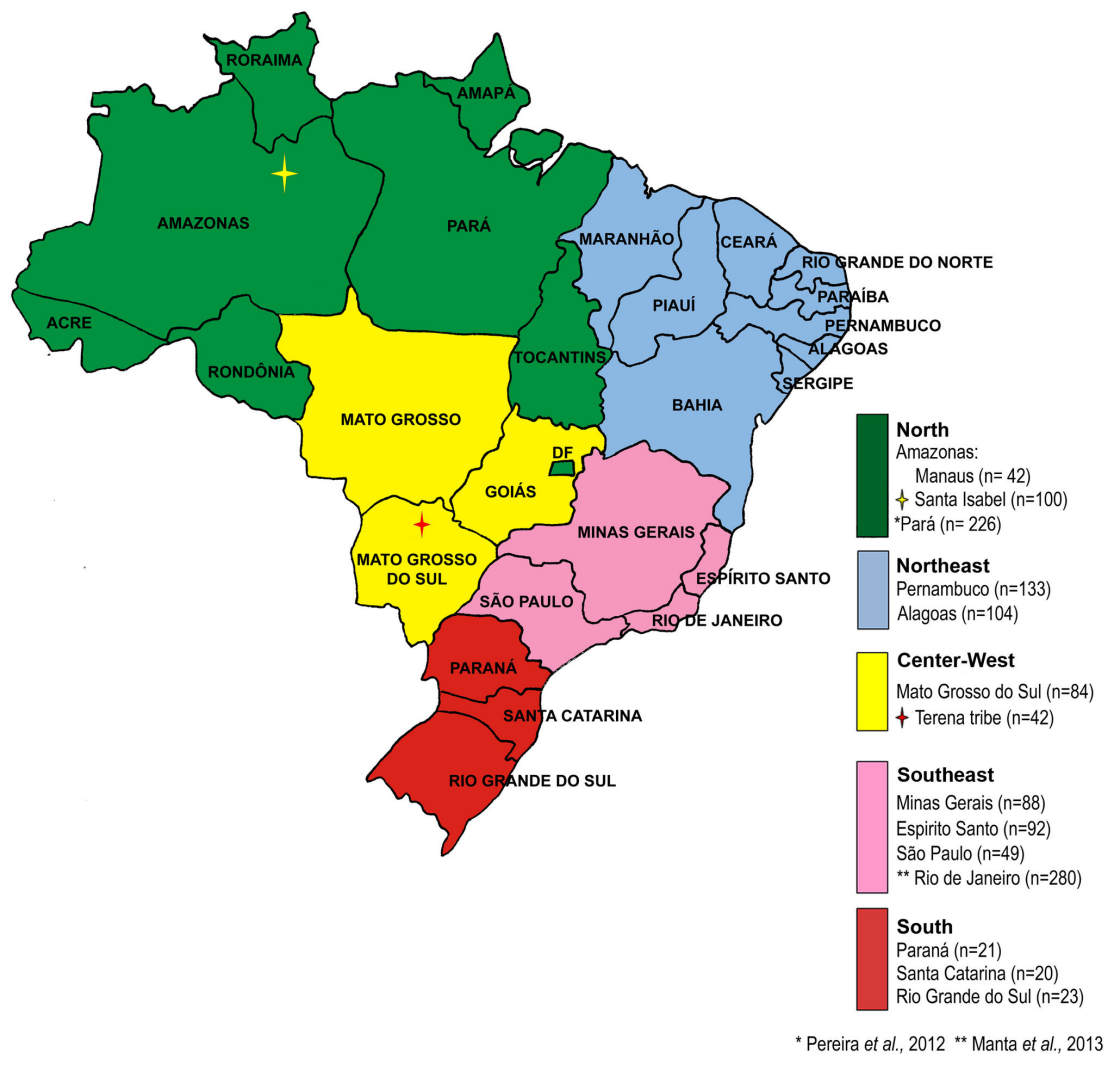
Map of Brazil showing the geographical location of the populations considered in the present study.

During this study, a total of 798 samples were collected from unrelated individuals from 12 different populations (see Figure 1 for the locations and number of samples), including (i) random samples from 10 out of 27 Brazilian Federative Units within Brazil’s five macro-regions, (ii) an Amerindian community, and (iii) a sample of Santa Isabel do Rio Negro (also known as simply “Santa Isabel”, the term adopted henceforth) that is a small admixed Amazonian population, which is quite isolated and known to have experienced a reduced exposure to European and African influxes.

The samples from Alagoas, Pernambuco, Mato Grosso do Sul, Minas Gerais, Espírito Santo, São Paulo, Paraná, Santa Cartarina and Rio Grande do Sul are random samples representing the state, excluding non-sampled Amerindian and Afro-descendant communities. The two samples from Amazonas do not represent the whole population of the state, since they have been collected just from individuals living in the rural area of Santa Isabel and in the city of Manaus. The samples from Rio Grande do Sul, Santa Catarina, Paraná, São Paulo and Minas Gerais were obtained from paternity casework at the DNA Diagnostic Laboratory (LDD). The samples from Pernambuco were collected at the Laboratory of Human Identification, University of Pernambuco, from cases of paternity investigation. The samples from Espírito Santo and Manaus were obtained from students and professors at the federal universities of Espírito Santo and Amazonas, respectively. The samples from Mato Grosso do Sul were obtained from students at the State school João Ponce de Arruda. The samples from Alagoas and Santa Isabel were selected from previous research projects belonging to healthy unrelated individuals used as controls in association studies, which involved the State hospitals as well as the Federal University of Alagoas and the Research Institute FIOCRUZ (Oswaldo Cruz Foundation). The Terena samples were those previously included in Manta et al. [22].

DNA was extracted following salting-out or Chelex standard protocols.

Our samples represent a random selection from the users of the services mentioned above (hospital, universities or paternity testing labs), and no information about the skin colour or ethnicity of the donors was considered for sample selection purposes. Other samples that were previously typed for the same set of markers were also used in data analyses, including Belém [3] and Rio de Janeiro [21] (see Figure 1). Additionally, to perform supervised ancestry analysis estimates, we used data available for HGDP-CEPH reference samples from African, European and Native American populations [3].

### Genetic markers and genotyping

A panel of 46 AIM-Indels was genotyped in a single multiplex PCR followed by capillary electrophoresis, according to the protocol described by Pereira et al. [3]. Dye-labelled amplified fragments were separated and detected using an ABI 3500 Genetic Analyzer (Life Technologies), and automated allele calls were obtained with GeneMapper v.4.1 (Life Technologies). Alleles’ nomenclature was according to Pereira et al. [3].

### Statistical analyses

Genetic diversity parameters, including the estimation of allele frequencies, observed and expected heterozygosities, Hardy-Weinberg exact tests and F_ST_ genetic distance analysis, were assessed by Arlequin v3.5 [23]. A multidimensional scaling (MDS) plot of the pairwise F_ST_ matrix was represented using the software STATISTICA v7.0 (Statsoft, Tulsa, Oklahoma; http://www.statsoft.com/).

The apportionment of genetic ancestral contributions from the different regions of Brazil was estimated using the STRUCTURE v2.3.3 software [24]. To estimate the ancestral membership proportions in the studied populations, a supervised analysis was performed using prior information on the geographic origin of the reference samples. Considering the historical formation of Brazil’s population, we assumed an essentially tri-hybrid contribution from Native Americans, Europeans and Africans (i.e., K=3) to the current genetic makeup of Brazilian populations. STRUCTURE runs consisted of 100,000 burnin steps followed by 100,000 Markov Chain Monte Carlo (MCMC) iterations. The option “*Use population Information to test for migrants*” was used with the Admixture model; allele frequencies were correlated and updated using only individuals with POPFLAG=1 (in this case, the HGDP-CEPH samples used as reference).

## Results

### Genetic characterisation of diversity in Brazil’s populations

The genotyping results for the 798 samples from 12 Brazilian populations are listed in Table S1. Allele frequencies and expected heterozygosities were estimated for the 46 AIM-Indels and are presented in Tables S2 and S3, respectively. As expected, the urban populations from Brazil show higher genetic variability than the parental populations because they harbour the contributions from three well-differentiated continental groups. The sample from the Terena native community has a similar level of diversity compared to the reference Native American sample, which is lower than that observed in urban populations. The same reduction in diversity was observed in the rural population of Santa Isabel, which is known to have been less exposed to European or African influx than the urban populations.

No statistically significant deviations from Hardy-Weinberg equilibrium expectations were found for the 46 loci in the 12 studied populations. Most Fisher’s exact test *p*-values were above 1%; lower values were only observed in 11 out of the 552 tests (0.00010≤ *p* ≤0.00764), but these are not significant when applying Bonferroni’s correction for multiple tests (significance level of 0.00009).

### Genetic distance analysis

**Figure 2:**
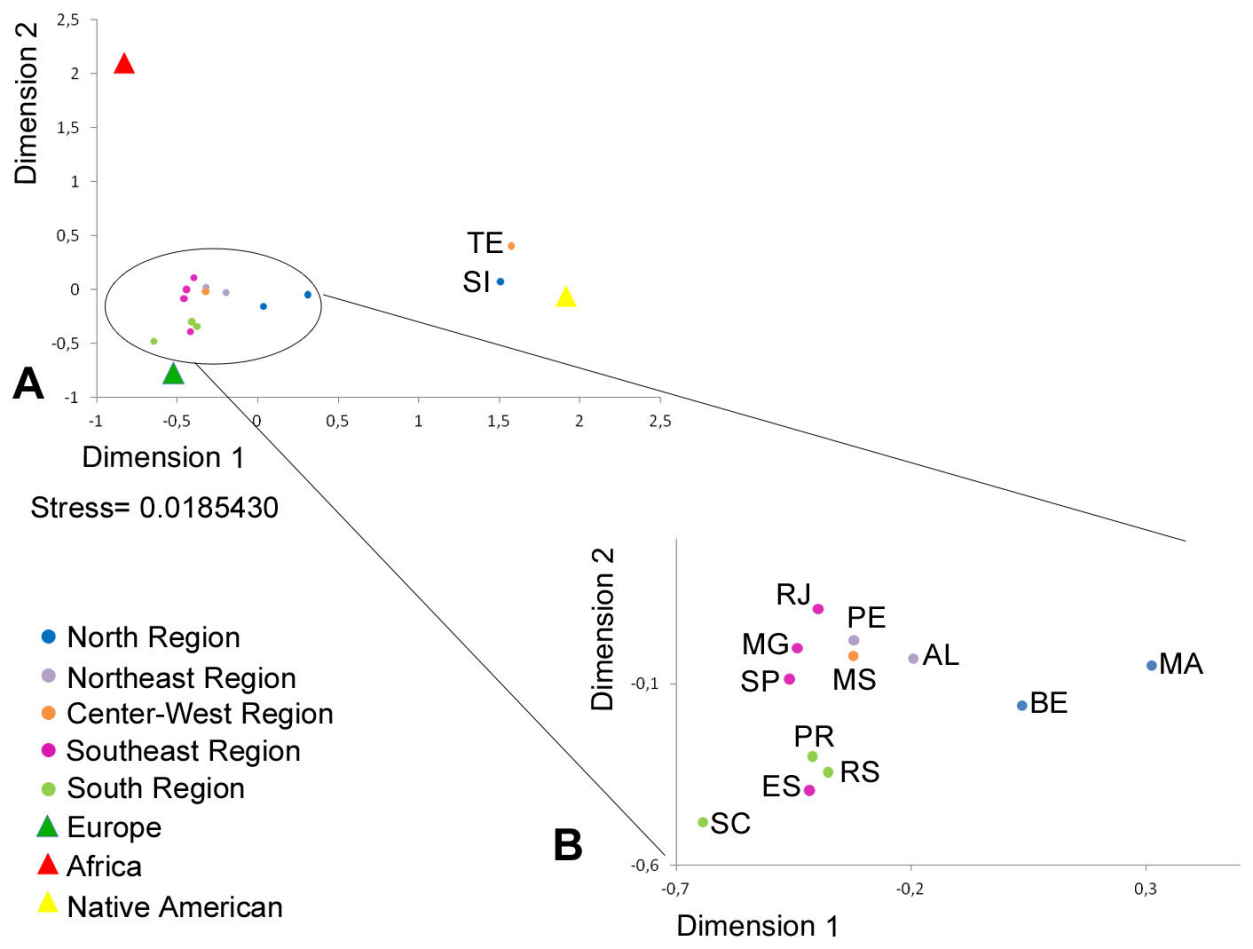
MDS plot of the F_ST_ pairwise genetic distances between the studied populations. (SI: Santa Isabel do Rio Negro; MA: Manaus; BE: Belém; PE: Pernambuco; AL: Alagoas; MS: Mato Grosso do Sul; TE: Terena; MG: Minas Gerais; ES: Espírito Santo; RJ: Rio de Janeiro; SP: São Paulo; PR: Paraná; SC: Santa Catarina; RS: Rio Grande do Sul.) F_ST_ genetic distances were assessed by Arlequin software and the MDS plot was represented using the software STATISTICA.

The data obtained for the 46 AIM-Indels in the 12 analysed samples were used to calculate the F_ST_ genetic distances between all population pairs together with the published data for the same markers in other Brazilian populations from Belém [3] and Rio de Janeiro [21] and from the parental African, European and Native American reference populations (Table S4). Figure 2 depicts the MDS plot of the pairwise F_ST_ matrix from Table S4.

Pairwise genetic distance analysis shows significant differentiation between most Brazilian samples and the ancestral populations, with the exception of two populations in the South (Santa Catarina and Paraná), which present a low genetic distance when compared with the Europeans. In most comparisons within each geographic region, no significant genetic distances were found between urban populations; significant genetic distances were only obtained between urban and non-urban samples, as well as between Espírito Santo and two other populations in the Southeast region.

Santa Isabel and Terena samples show the lowest genetic distances to the Native Americans. The remaining Brazilian populations are all closer to the Europeans, although the positions in the MDS plot of the Northern populations of Manaus and Belém indicate a significant Native American contribution to these populations (Figure 2). In general, genetic distances to Native Americans are lower for populations in the North and higher for populations in the South. Conversely, the lowest genetic distances to Europeans are noted in Southern populations. Finally, the genetic composition of the Northeast, Center-West and Southeast regions is very similar, with slightly lower genetic distances to the Africans when compared to populations from the North or the South. The southeastern population from Espírito Santo is an exception and appears closer to the South, showing a lower genetic distance to Europeans than the other samples from the Southeast region.

### Interethnic admixture analysis

**Figure 3:**
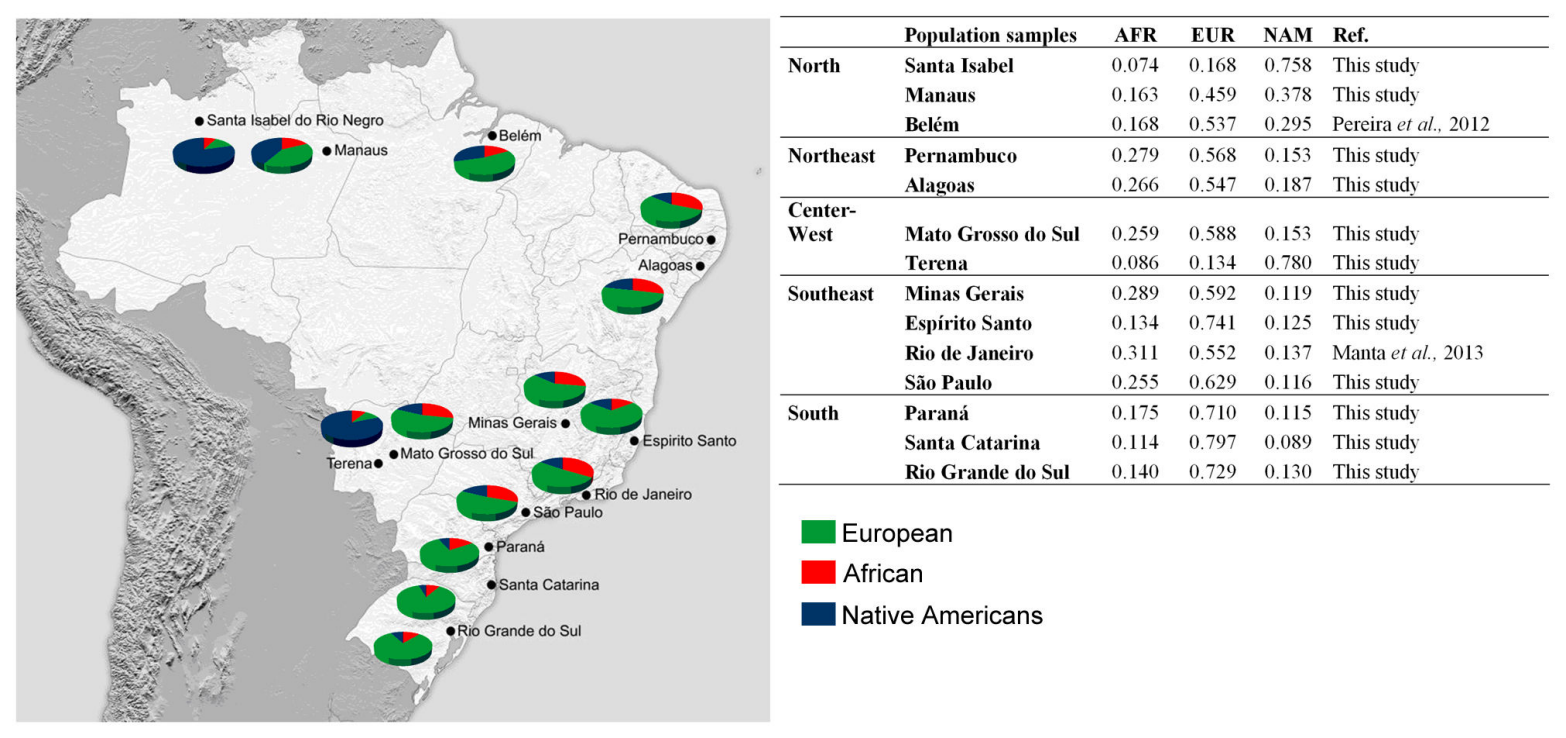
Average ancestral membership proportions obtained for the Brazilian testing populations using 46 AIM-Indels. Estimates were obtained using STRUCTURE, for the following options: k=3; 100,000 burnin steps followed by 100,000 MCMC iterations; Admixture model (*“Use population Information to test for migrants”*); and allele frequencies were correlated and updated using only individuals with POPFLAG=1.

The software STRUCTURE was used to estimate the ancestry membership proportions in populations from different regions in Brazil. The ancestry analyses were based on the genotypic data generated in the present study as well as previously published data for HGDP-CEPH reference samples from Africans, Europeans and Native Americans and the Brazilian populations of Belém and Rio de Janeiro [3,21]. The ancestry estimates obtained for all populations are shown in Figure 3.

The results are consistent with the genetic distance analysis. The Native American group of Terena and the non-urban Amazonian population of Santa Isabel exhibit a Native American contribution to their genetic pool that is above 75%.

European ancestry is the most prevalent in all urban populations, achieving the highest values (above 70%) in the three populations from the South. The populations in the North consist of a significant proportion of Native American ancestry that is approximately twice as high as that of African ancestry. Conversely, in the Northeast, Center-West and Southeast, the African contribution was the second most prevalent. As previously corroborated by the results of genetic distance analysis, the sample from Espírito Santo better fits the genetic profile of the populations in the South than those in the Southeast region.

**Figure 4:**
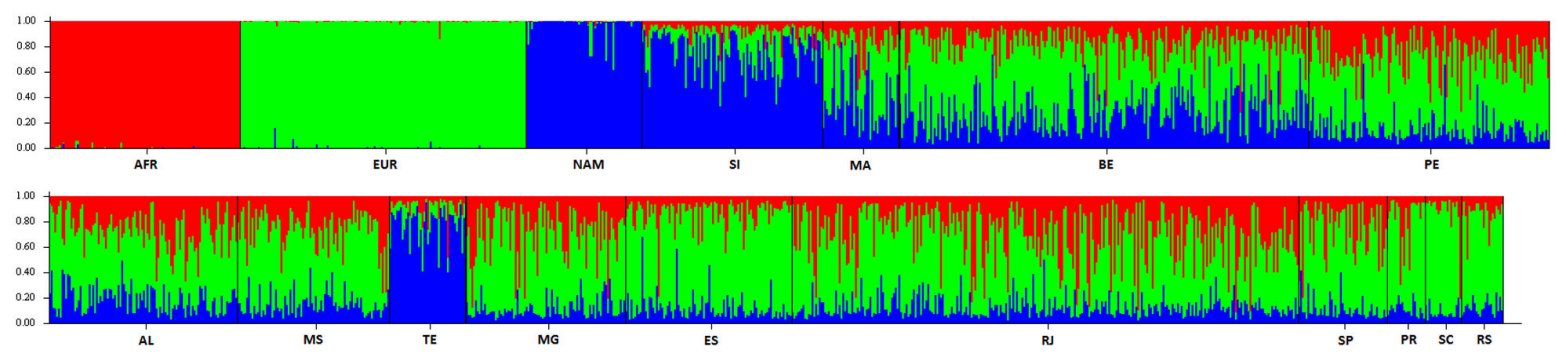
Individual ancestry estimates obtained for the HGDP-CEPH reference samples and individuals tested from Brazilian populations using 46 AIM-Indels (AFR: Africa; EUR: Europe; NAM: Native American; SI: Santa Isabel do Rio Negro; MA: Manaus; BE: Belém; PE: Pernambuco; AL: Alagoas; MS: Mato Grosso do Sul; TE: Terena; MG: Minas Gerais; ES: Espírito Santo; RJ: Rio de Janeiro; SP: São Paulo; PR: Paraná; SC: Santa Catarina; RS: Rio Grande do Sul). Ancestry estimates were obtained using STRUCTURE, for the following options: k=3; 100,000 burnin steps followed by 100,000 MCMC iterations; Admixture model (*“Use population Information to test for migrants”*); and allele frequencies were correlated and updated using only individuals with POPFLAG=1.

At an intrapopulation level, heterogeneity is observed in all urban populations (Figure 4), with a very wide range of variation of ancestry proportions between individuals within populations. Nevertheless, individuals with a high proportion of Native American ancestry are only found in the samples from Terena and Santa Isabel.

## Discussion

A panel of 46 AIM-Indels was recently described by Pereira et al. [3] to show marked allele frequency differentiation among main human population groups and proved to be highly informative for inferring ancestry. This panel of ancestry-informative Indels was used to characterise and compare the genetic composition of over 1,300 individuals from 14 populations among the five geopolitical regions in which Brazil is usually subdivided; to our knowledge, this represents the most comprehensive nationwide ancestry assessment undertaken using autosomal AIMs.

In the present work, a north-south decreasing F_ST_ gradient was noticeable between the Brazilian and the European samples, which appears to be directly correlated with the increasing European membership proportions depicted in the ancestry analysis. On the other hand, the lower distances between Native Americans and the populations from the North are in agreement with the slightly higher Native American composition that was detected in those populations. Additionally, in populations from the Northeast, Center-West and Southeast, lower F_ST_ genetic distances were detected when compared with Africans together with higher African ancestry proportions. In general, the genetic distances are low among samples from large urban Brazilian populations, but in many cases, they are statistically significant (Table S4). The low differentiation associated with a high European ancestry that is observed in large urban populations cannot be extrapolated to smaller and more isolated rural populations or to the significant number of Native or Afro-Brazilian communities throughout the nation. A good example of this is the ancestry pattern observed in the three populations located in the North region that were included in this study. In these populations, the proportion of Native American ancestry increases in the smaller and more isolated populations. Indeed, it is almost 10% higher in Manaus than in Belém, and much higher in Santa Isabel than in Belém or Manaus. Different native groups from Amazonia have contributed to the population of Santa Isabel, which is indeed highly ethnically admixed and is not a native community established by a single ethno-linguistic group as is the case for Terena in Mato Grosso. Nevertheless, these two populations harbour similar African, European and Native American contributions, which emphasises the importance of geographic isolation and cultural barriers in developing the substructure within the main geopolitical regions.

These findings are consistent with the demographic patterns depicted throughout the country ( [9]; Brazilian Institute of Geography and Statistics (IBGE); http://www.ibge.gov.br). However, IBGE statistics are based on colour classification, which precludes a strict correlation between demography and genetics. Indeed, past investigations have shown a weak correlation between skin colour or self-declared ethnicity and the genetic ancestry of individuals obtained from the characterisation of Ancestry Informative Markers (e.g., [25,26,27,28,29]).

### Demographic significance of the selected samples

According to the last IBGE population survey in 2012, Brazil has approximately 194 million inhabitants distributed among the five geographic regions (http://www.ibge.gov.br/home/estatistica/populacao/estimativa2012). Most populations are highly admixed, and approximately 84% inhabitants live in large urban cities. In urban areas, only 0.2% of the population is Native American. The remaining 16% lives in rural areas, where Native Americans represent almost 2% of the population. Additionally, in rural areas, there are remnants of communities that were originally formed by fugitive slaves, known as Quilombos. These Afro-Brazilian communities are not demographically very significant, although they are numerous. Of the 1,826 existing communities that are dispersed all over the country, only 190 are currently officially recognised, consisting of just 11,946 families. Based on data from the IBGE, these communities occupy 0.12% of the national territory.

To maximise the collection of genetic diversity present in each region, we have selected samples from the main urban cities, where people are more concentrated and where the development of nearby small rural communities tends to genetically contribute. Samples from a native community in Mato Grosso do Sul and from a small rural population in Amazonia were also included in our study because Native American communities in Brazil represent a non-negligible fraction of the population.

Overall, considering the urban population samples that we studied representative of their States and taking into account the number of inhabitants in the IBGE demographic census (see Table S5 for details), we attained near complete coverage in the South and Southeast regions (with the exception of Native and Afro-descendant communities), 70% coverage in the North and only ~20% in the Northeast and the less populous Central-West regions. As a whole, the 14 populations that were analysed in the present study represent approximately 70% of the global Brazilian population.

### Genetic ancestry of the Brazilian populations revisited

Many attempts have been made to determine the ethnic diversity of Brazil, to infer patterns of variation throughout the country and to note differences among communities with particular histories. Furthermore, many different types of markers have been used to pursue this objective.

Studies on uniparental markers were especially useful to discern male- and female- specific features. Nevertheless, for most studies on Y chromosome diversity in Brazilian populations, only Y-STRs (e.g., [19,30,31,32,33]) or just a restricted number of SNP markers (e.g., [26,34,35]) were studied, which often limited the accuracy of ancestry estimates derived from the three continents. Regarding the studies on the mtDNA variability in Brazilian populations, a very high percentage has been dedicated to small communities of Native Americans and Afro-descendants (e.g., [15]), but only few describe the composition of urban admixed populations [16,36]. In general, lineage markers have shown that in almost all Brazilian populations studied until now, the admixture was characterised by an asymmetric mating pattern occurring preferentially between European men and Native American or African women (e.g., [37,38]). In the Afro-descendant communities (known as Quilombos) in Amazonia and Maranhão, an asymmetric mating pattern was also observed between African men and Native American women (e.g., [15,39]).

Various studies were undertaken using autosomal markers, including blood groups and proteins and, more recently, polymorphisms representing different types of DNA variation, namely, STRs, SNPs and Indels (e.g., [3,11,12,20,40]). In general, they indicated a stronger Native American influence in the north. Significant African input in the northeast region was demonstrated, whereas the European contribution was rather global, following an increasing north-south gradient.

**Figure 5:**
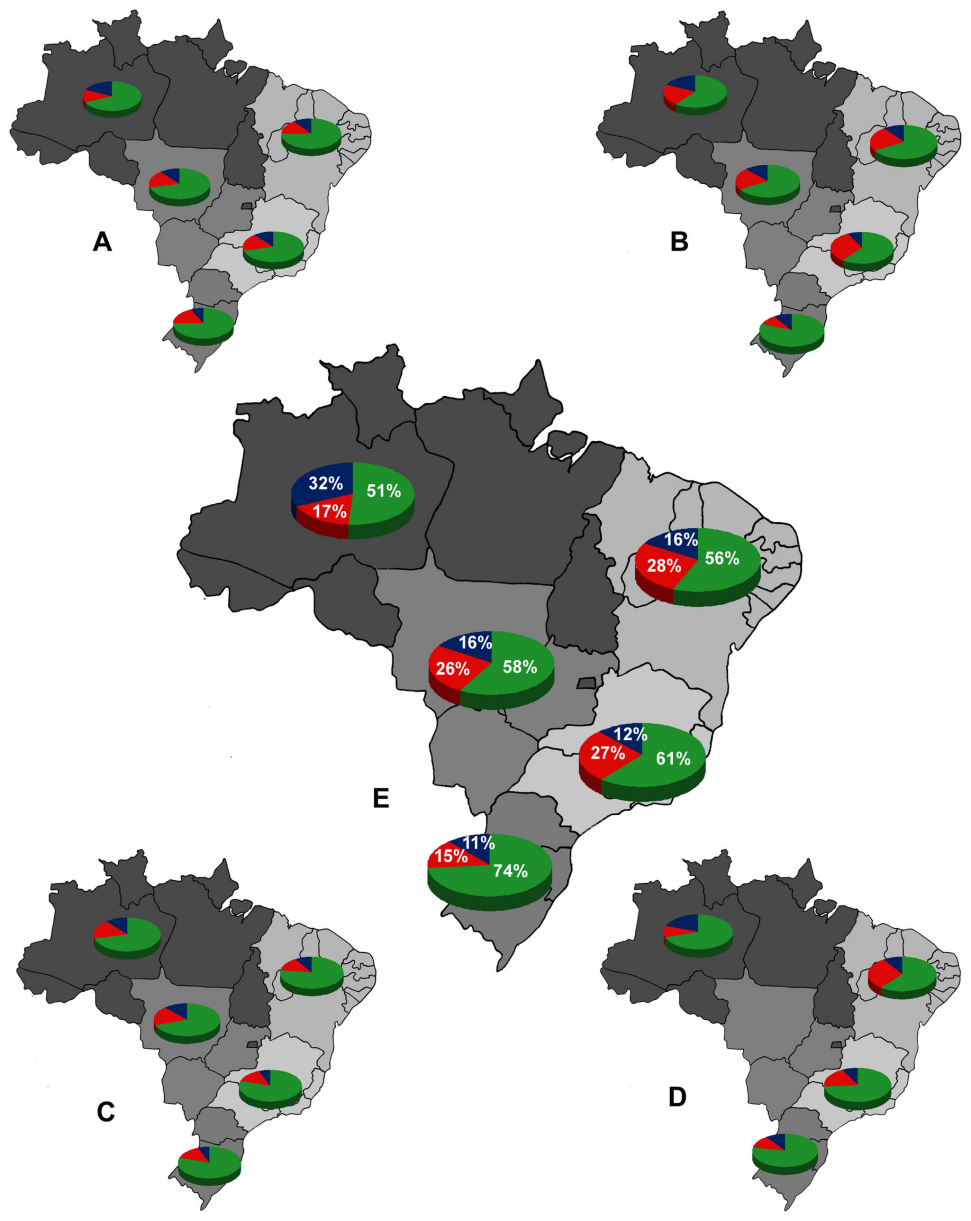
Comparison of the European, African and Native American ancestry estimates obtained in the present work and in previous studies for the five regions of Brazil. In the present work, the overall values indicated for each region are a weighted average of ancestry estimates of the population samples studied in that region considering their respective representation among inhabitants. A: Callegari-Jacques et al. [10]; B: Godinho et al. [11]; C: Lins et al. [20]; D: Pena et al. [40]; E: this study.

The same pattern of genetic variation throughout the country was observed in the present study, although we could find slight differences between our estimates and those from Callegari-Jacques et al. [10], Godinho et al. [11], Lins et al. [20] and Pena et al. [40] (see Figure 5). The observed discrepancies can be attributed to the number and/or type of markers and the different sampling strategies that were used. Unfortunately, such conceptual particularities in the design of each study that we reviewed hindered a direct and more comprehensive comparison between ancestry studies performed in Brazil. Nonetheless, a global overview of all studies shows a major European contribution across all regions of the country, despite some variation in their estimations. Furthermore, a general concordance extends to the regional level. The Northeast, Center-West, Southeast and South all reveal an admixed pattern of mainly European ancestry followed by African and Native American genetic influences. The exception occurs in the North, where the Native American membership proportion was higher than the African proportion in some studies [10,40, this study] compared with others [11,20]. Our study had the peculiarity of yielding lower European ancestry estimates while at the same time depicting stronger Native American contributions. As already noted, some of these differences may be due to the number and/or type of markers used and different sampling strategies. We highlight the fact that we have used carefully selected AIMs to capture the greatest amount of genetic differentiation among ancestral parental groups; we have also collected the greatest quantity of samples involved in a single ancestry assessment study in Brazil.

### The relevance of sampling strategies

In any genetic study, samples always represent a reference group and not a real population, unless they are absolutely randomly collected. Nevertheless, perfectly random population samples are not necessarily or not always the most useful ones, and sampling strategies must reflect the objective of a research project or application. For example, samples that include information on the grandparents’ birthplace, although very useful to learn about the history of a population, do not represent the actual living or residential population; thus, they have limited value for forensic or medical purposes.

Most studies on the ethnicity of South American populations aim to evaluate admixture processes occurring in separate groups within a population. Nevertheless, the classification of groups is complicated, and different studies can use different criteria (e.g., self-declaration or ethnic classification by the researchers). Moreover, phenotypic classifications are usually subjective, and a person can self-report himself into different groups at different time points. Alternatively, people can classify others in different ways (e.g., [26,28,41,42]). A poor correlation between phenotypic/ethnic classifications and genetic ancestry has been found in most studies of Brazilian population groups, which prevents extrapolation of results based on some groups to the overall population. For instance, it was demonstrated that groups with equivalent self-reported proportions, but from populations with different levels of urbanisation [28] or from different regions of Brazil [40], can have different genetic ancestry profiles.

For the reasons mentioned above, the samples included in our study were randomly selected from unrelated individuals living in one of the targeted populations without using any criteria related to individual ethnicity.

In many publications that evaluate population ancestry, a frequent issue is the nature of the sampling. Samples are frequently selected from hospitals, universities, genetic diagnostic units, or other entities that do not necessarily represent the whole population, as the European, Native American and African genetic ancestry proportions can be affected by educational or socioeconomic status (e.g., [10,37]). This was also the case in the present study, and we believe, therefore, that this was the main reason for the discrepant results we found for the population of Espírito Santo. The samples had been collected from students and professors in a university, which was pointed out before to lead to an increased European proportion (e.g., [10]). For that reason, we have to carefully interpret the lower African and higher European ancestries that we found in Espírito Santo in comparison to the other three populations from the Southeast region, namely Minas Gerais, Rio de Janeiro and São Paulo. Thus, it would be desirable to better scrutinise the ethnic substructure of the Southeast by studying other source samples from Espírito Santo. The sample from Manaus was also collected in a University and, therefore, we cannot exclude a slightly higher European contribution; although this is not apparent when comparing our results with previous estimates (Figure 5).

The consistency of the results obtained among populations and within groups of populations, in addition to the expectations based on the available genetic and demographic data, precludes any significant distortion of the results obtained in the remaining populations we have studied. These samples are from paternity investigation cases preformed by private or judicial request, covering, therefore, people from both the lower and the higher social status that are living in the concerned states.

## Conclusion

Overall, the present results highlight the considerable amount of ethnic admixture that occurred throughout the country and that the current Brazilian population is significantly differentiated from the ancestral Native American, European and African populations that have been incorporated into its genepool during the last five centuries. The arrival of the Europeans stands out as the major event that shaped the genetic landscape of Brazil, which is especially evident in urban populations. Although demographically less representative of Brazil and spread throughout the country, there are still many rural populations and native communities that have maintained a strong Native American background. The African legacy is also a hallmark of all Brazilian populations, and in the present study represents more than 25% of the genetic makeup of most urban populations with the exception of the Northern and Southern regions (from 11 to 18%).

Apart from the variation found among populations throughout the country, our study also underscores the tremendous diversity that can be found among individuals from the same population in terms of the three different ethnic contributions (a wide spectrum can be observed, ranging from a more or less balanced contribution from two or three sources to an almost single source ancestry).

In accordance with previous studies, our results emphasise the heterogenity of the Brazilian population from within and among populations, which justifies the need for additional studies involving more markers in new populations to allow an accurate assessment of the genetic ancestry for each subpopulation.

## Supporting Information

Table S1 **List of genotypes found in 12 different populations included in the present work as well as data from Rio de Janeiro, Brazil, previously published by Manta et al., 2013.**
(XLS)

Table S2 **Allele frequencies for 46 AIM-Indels in twelve Brazilian populations.**
(XLS)

Table S3 **Gene diversities for 46 AIM-Indels in twelve Brazilians populations.**
(XLS)

Table S4 **Genetic distances (F_ST_) between African, European, Native American and Brazilian populations (lower diagonal) and corresponding non-differentiation P values (upper diagonal).**
(XLS)

Table S5 **Demographic significance of sampled populations based in 2010 IBGE data for Brazilian Regions and States.**
(PDF)

## References

[1] Parra EJ, Marcini A, Akey J, Martinson J, Batzer MA et al. (1998) Estimating African American admixture proportions by use of population-specific alleles. Am J Hum Genet 63: 1839-1851. doi:10.1086/302148. PubMed: 9837836. 9837836 10.1086/302148 PMC1377655

[2] Kosoy R, Nassir R, Tian C, White PA, Butler LM et al. (2009) Ancestry informative marker sets for determining continental origin and admixture proportions in common populations in America. Hum Mutat 30: 69-78. doi:10.1002/humu.20822. PubMed: 18683858. 18683858 10.1002/humu.20822 PMC3073397

[3] Pereira R, Phillips C, Pinto N, Santos C, Santos SEB et al. (2012) Straightforward inference of ancestry and admixture proportions through ancestry-informative insertion deletion multiplexing. PLOS ONE 7: e29684. doi:10.1371/journal.pone.0029684. PubMed: 22272242. 22272242 10.1371/journal.pone.0029684 PMC3260179

[4] Pritchard JK, Donnelly P (2001) Case-control studies of association in structured or admixed populations. Theor Popul Biol 60: 227-237. doi:10.1006/tpbi.2001.1543. PubMed: 11855957. 11855957 10.1006/tpbi.2001.1543

[5] Zembrzuski VM, Callegari-Jacques SM, Hutz MH (2006) Application of an African Ancestry Index as a genomic control approach in a Brazilian population. Ann Hum Genet 70: 822-828. doi:10.1111/j.1469-1809.2006.00270.x. PubMed: 17044857. 17044857 10.1111/j.1469-1809.2006.00270.x

[6] Tian C, Gregersen PK, Seldin MF (2008) Accounting for ancestry: population substructure and genome-wide association studies. Hum Mol Genet 17: R143-R150. doi:10.1093/hmg/ddn268. PubMed: 18852203. 18852203 10.1093/hmg/ddn268 PMC2782357

[7] Amorim CEG, Falcão-Alencar G, Godinho NMO, Diniz MECG, Gontijo CC et al. (2009) Forensic application of an individual ancestry index in Brazilian populations. Forensic Science International: Genetics Supplement Series 2: 479-480.

[8] Kayser M, de Knijff P (2011) Improving human forensics through advances in genetics, genomics and molecular biology. Nat Rev Genet 12: 179-192. doi:10.1038/nrg2952. PubMed: 21331090. 21331090 10.1038/nrg2952

[9] Brasileirode Instituto Geografia e Estatística (2000) Brasil : 500 anos de povoamento/Brazil -500 years of settlement. Rio de Janeiro: IBGE: 232.

[10] Callegari-Jacques SM, Grattapaglia D, Salzano FM, Salamoni SP, Crossetti SG et al. (2003) Historical genetics: spatiotemporal analysis of the formation of the Brazilian population. Am J Hum Biol 15: 824-834. doi:10.1002/ajhb.10217. PubMed: 14595874. 14595874 10.1002/ajhb.10217

[11] Godinho NMO, Gontijo CC, Diniz MECG, Falcão-Alencar G, Dalton GC et al. (2008) Regional patterns of genetic admixture in South America. Forensic Science International: Genetics Supplement Series 1: 329-330.

[12] Santos NP, Ribeiro-Rodrigues EM, Ribeiro-Dos-Santos AK, Pereira R, Gusmão L et al. (2010) Assessing individual interethnic admixture and population substructure using a 48-insertion-deletion (INSEL) ancestry-informative marker (AIM) panel. Hum Mutat 31: 184-190. doi:10.1002/humu.21159. PubMed: 19953531. 19953531 10.1002/humu.21159

[13] Schneider H, Salzano FM (1979) Gm allotypes and racial admixture in two Brazilian populations. Hum Genet 53: 101-105. doi:10.1007/BF00289458. PubMed: 295040. 295040 10.1007/BF00289458

[14] Ribeiro-dos-Santos AK, Pereira JM, Lobato MR, Carvalho BM, Guerreiro JF et al. (2002) Dissimilarities in the process of formation of Curiau, a semi-isolated Afro-Brazilian population of the Amazon region. Am J Hum Biol 14: 440-447. doi:10.1002/ajhb.10059. PubMed: 12112565. 12112565 10.1002/ajhb.10059

[15] Bortolini MC, Da Silva Junior WA, De Guerra DC, Remonatto G, Mirandola R et al. (1999) African-derived South American populations: A history of symmetrical and asymmetrical matings according to sex revealed by bi- and uni-parental genetic markers. Am J Hum Biol 11: 551-563. doi:10.1002/(SICI)1520-6300(1999)11:4. PubMed: 11533975. 11533975 10.1002/(SICI)1520-6300(1999)11:4<551::AID-AJHB15>3.0.CO;2-Z

[16] Alves-Silva J, da Silva Santos M, Guimarães PE, Ferreira AC, Bandelt HJ et al. (2000) The ancestry of Brazilian mtDNA lineages. Am J Hum Genet 67: 444-461. doi:10.1086/303004. PubMed: 10873790. 10873790 10.1086/303004 PMC1287189

[17] Sans M (2000) Admixture studies in Latin America: from the 20th to the 21st century. Hum Biol 72: 155-177. PubMed: 10721616. 10721616

[18] Silva DA, Carvalho E, Costa G, Tavares L, Amorim A et al. (2006) Y-chromosome genetic variation in Rio de Janeiro population. Am J Hum Biol 18: 829-837. doi:10.1002/ajhb.20567. PubMed: 17039481. 17039481 10.1002/ajhb.20567

[19] Francez PA, Ramos LP, Palha TJ, Santos SEB (2012) Haplotype diversity of 17 Y-STR loci in an admixed population from the Brazilian Amazon. Genet Mol Biol 35: 45-52. doi:10.1590/S1415-47572011005000061. PubMed: 22481873. 22481873 10.1590/s1415-47572011005000061 PMC3313515

[20] Lins TC, Vieira RG, Abreu BS, Grattapaglia D, Pereira RW (2010) Genetic composition of Brazilian population samples based on a set of twenty-eight ancestry informative SNPs. Am J Hum Biol 22: 187-192. PubMed: 19639555. 19639555 10.1002/ajhb.20976

[21] Manta FSN, Pereira R, Caiafa A, Silva DA, Gusmão L et al. (2013) Analysis of genetic ancestry in the admixed Brazilian population from Rio de Janeiro using 46 autosomal ancestry-informative indel markers. Ann Hum Biol 40: 94-98. PubMed: 23151124. 23151124 10.3109/03014460.2012.742138

[22] Manta F, Caiafa A, Pereira R, Silva D, Amorim A et al. (2012) Indel markers: genetic diversity of 38 polymorphisms in Brazilian populations and application in a paternity investigation with post mortem material. Forensic Sci Int Genet 6: 658-661. doi:10.1016/j.fsigen.2011.12.008. PubMed: 22277257. 22277257 10.1016/j.fsigen.2011.12.008

[23] Excoffier L, Lischer HE (2010) Arlequin suite ver 3.5: a new series of programs to perform population genetics analyses under Linux and Windows. Mol Ecol Resour 10: 564-567. doi:10.1111/j.1755-0998.2010.02847.x. PubMed: 21565059. 21565059 10.1111/j.1755-0998.2010.02847.x

[24] Pritchard JK, Stephens M, Donnelly P (2000) Inference of population structure using multilocus genotype data. Genetics 155: 945-959. PubMed: 10835412. 10835412 10.1093/genetics/155.2.945 PMC1461096

[25] Parra EJ, Kittles RA, Shriver MD (2004) Implications of correlations between skin color and genetic ancestry for biomedical research. Nat Genet 36: S54-S56. doi:10.1038/ng1440. PubMed: 15508005. 15508005 10.1038/ng1440

[26] Marrero AR, Das Neves Leite FP, De Almeida Carvalho B, Peres LM, Kommers TC et al. (2005) Heterogeneity of the genome ancestry of individuals classified as White in the state of Rio Grande do Sul, Brazil. Am J Hum Biol 17: 496-506. doi:10.1002/ajhb.20404. PubMed: 15981186. 15981186 10.1002/ajhb.20404

[27] Leite TK, Fonseca RM, de França NM, Parra EJ, Pereira RW (2011) Genomic ancestry, self-reported "color" and quantitative measures of skin pigmentation in Brazilian admixed siblings. PLOS ONE 6: e27162. doi:10.1371/journal.pone.0027162. PubMed: 22073278. 22073278 10.1371/journal.pone.0027162 PMC3206941

[28] Lins TC, Vieira RG, Abreu BS, Gentil P, Moreno-Lima R et al. (2011) Genetic heterogeneity of self-reported ancestry groups in an admixed Brazilian population. J Epidemiol 21: 240-245. doi:10.2188/jea.JE 20100164. PubMed: 21498954 21498954 10.2188/jea.JE20100164 PMC3899415

[29] Cardena MMSG, Ribeiro-dos-Santos Â, Santos S, Mansur AJ, Pereira AC et al. (2013) Assessment of the Relationship between Self-Declared Ethnicity, Mitochondrial Haplogroups and Genomic Ancestry in Brazilian Individuals. PLOS ONE 8: e62005. doi:10.1371/journal.pone.0062005. PubMed: 23637946. 23637946 10.1371/journal.pone.0062005 PMC3634831

[30] Leite FP, Callegari-Jacques SM, Carvalho BA, Kommers T, Matte CH et al. (2008) Y-STR analysis in Brazilian and South Amerindian populations. Am J Hum Biol 20: 359-363. doi:10.1002/ajhb.20702. PubMed: 18161040. 18161040 10.1002/ajhb.20702

[31] Palha TJ, Ribeiro-Rodrigues EM, Ribeiro-dos-Santos A, Guerreiro JF, Moura LS et al. (2011) Male ancestry structure and interethnic admixture in African-descent communities from the Amazon as revealed by Y-chromosome Strs. Am J Phys Anthropol 144: 471-478. doi:10.1002/ajpa.21436. PubMed: 21302273. 21302273 10.1002/ajpa.21436

[32] Palha TJ, Gusmão L, Ribeiro-Rodrigues E, Guerreiro JF, Ribeiro-Dos-Santos A et al. (2012) Disclosing the genetic structure of Brazil through analysis of male lineages with highly discriminating haplotypes. PLOS ONE 7: e40007. doi:10.1371/journal.pone.0040007. PubMed: 22808085. 22808085 10.1371/journal.pone.0040007 PMC3393733

[33] Wiezel CE, Luizon MR, Sousa SM, Santos LM, Muniz YC et al. (2013) Y-Linked microsatellites in Amazonian Amerindians applied to ancestry estimates in Brazilian Afro-derived populations. Am J Hum Biol 25: 313-317. doi:10.1002/ajhb.22361. PubMed: 23348861. 23348861 10.1002/ajhb.22361

[34] Santos SEB, Rodrigues JD, Ribeiro-dos-Santos AK, Zago MA (1999) Differential contribution of indigenous men and women to the formation of an urban population in the Amazon region as revealed by mtDNA and Y-DNA. Am J Phys Anthropol 109: 175-180. doi:10.1002/(SICI)1096-8644(199906)109:2. PubMed: 10378456. 10378456 10.1002/(SICI)1096-8644(199906)109:2<175::AID-AJPA3>3.0.CO;2-#

[35] Hünemeier T, Carvalho C, Marrero AR, Salzano FM, Junho Pena SD et al. (2007) Niger-Congo speaking populations and the formation of the Brazilian gene pool: mtDNA and Y-chromosome data. Am J Phys Anthropol 133: 854-867. doi:10.1002/ajpa.20604. PubMed: 17427922. 17427922 10.1002/ajpa.20604

[36] Barbosa AB, da Silva LA, Azevedo DA, Balbino VQ, Mauricio-da-Silva L (2008) Mitochondrial DNA control region polymorphism in the population of Alagoas state, north-eastern Brazil. J Forensic Sci 53: 142-146. doi:10.1111/j.1556-4029.2007.00619.x. PubMed: 18279250. 18279250 10.1111/j.1556-4029.2007.00619.x

[37] Salzano FM (2004) Interethnic variability and admixture in Latin America--social implications. Rev Biol Trop 52: 405-415. PubMed: 17361535. 17361535 10.15517/rbt.v1i2.15273

[38] Pena SD, Bastos-Rodrigues L, Pimenta JR, Bydlowski SP (2009) DNA tests probe the genomic ancestry of Brazilians. Braz J Med Biol Res 42: 870-876. doi:10.1590/S0100-879X2009005000026. PubMed: 19738982. 19738982 10.1590/s0100-879x2009005000026

[39] Carvalho BM, Bortolini MC, Santos SEBd, Ribeiro-dos-Santos ÂKC (2008) Mitochondrial DNA mapping of social-biological interactions in Brazilian Amazonian African-descendant populations. Genet Mole Biol 31: 12-22.

[40] Pena SD, Di Pietro G, Fuchshuber-Moraes M, Genro JP, Hutz MH et al. (2011) The genomic ancestry of individuals from different geographical regions of Brazil is more uniform than expected. PLOS ONE 6: e17063. doi:10.1371/journal.pone.0017063. PubMed: 21359226. 21359226 10.1371/journal.pone.0017063 PMC3040205

[41] Domingues PM, Gusmão L, Silva DA, Amorim A, Pereira RW et al. (2007) Sub-Saharan Africa descendents in Rio de Janeiro (Brazil): population and mutational data for 12 Y-STR loci. Int J Leg Med 121: 238-241. doi:10.1007/s00414-007-0154-x. PubMed: 17334737. 10.1007/s00414-007-0154-x 17334737

[42] Guerreiro-Junior V, Bisso-Machado R, Marrero A, Hünemeier T, Salzano FM et al. (2009) Genetic signatures of parental contribution in black and white populations in Brazil. Genet Mol Biol 32: 1-11. doi:10.1590/S1415-47572009005000001. PubMed: 21637639. 21637639 10.1590/S1415-47572009005000001 PMC3032968

