# Supplementary material for: Revisiting the Genetic Ancestry of Brazilians Using Autosomal AIM-Indels
Source: PLoS One. 2013 Sep 20;8(9):e75145. doi: 10.1371/journal.pone.0075145 (PMC3779230; doi:10.1371/journal.pone.0075145)
Supplement: Table S5 — Demographic significance of sampled populations based in 2010 IBGE data for Brazilian Regions and States. (PDF) [file pone.0075145.s005.pdf]

Table S5. Demographic significance of the sampled populations based in 2010 IBGE data for Brazilian Regions and States.

	Total population (IBGE)	Population representation	(%)
Santa Isabel (Amazonas)	18146	11083182	70%
Manaus (Amazonas)	3483985		
Belém (Pará)	7581051		
**North**	15864454		
Pernanbuco	8796448	11916942	22%
Alagoas	3120494		
**Northeast**	53081950		
Mato Grosso do Sul	2449024	2472024	18%
Terena	23000		
**Center-West**	14058094		
Minas Gerais	19597330	80364410	100% *
Espírito Santo	3514952		
Rio de Janeiro	15989929		
São Paulo	41262199		
**Southeast**	80364410		
Paraná	10444526	27386891	100% *
Santa Catarina	6248436		
Rio Grande do Sul	10693929		
**South**	27386891		
**Brasil**	190755799	133223449	69%

* excluding non-sampled Amerindian and Afro-descendant communities
